# Mitophagy in Age-Dependent Neurodegeneration

**DOI:** 10.32607/actanaturae.27674

**Published:** 2025

**Authors:** V. S. Sukhorukov, A. V. Egorova, A. S. Romanenko, M. S. Ryabova, A. P. Krasilnikova

**Affiliations:** Russian Center of Neurology and Neurosciences, Moscow, 125367 Russia; Pirogov Russian National Research Medical University, Moscow, 117513 Russia; M.V. Lomonosov Moscow State University, Moscow, 119234 Russia

**Keywords:** mitophagy, mitochondria, aging, Alzheimer’s disease, Parkinson’s disease

## Abstract

Mitochondrial dysfunction is one of the pathogenetic mechanisms of neuronal damage during aging. The high energy dependence of neurons makes them particularly vulnerable to age-related changes accompanied by oxidative stress and impaired energy metabolism. The maintenance of a pool of functional mitochondria is regulated by mitophagy, which ensures the utilization of damaged organelles, thereby preventing the progression of mitochondrial dysfunction. Brain aging is accompanied by a reduced level of activity of metabolic processes, aggravated mitochondrial dysfunction, and an increased risk of developing neurodegenerative diseases such as Alzheimer’s disease and Parkinson’s disease. This review highlights the molecular and signaling pathways of mitophagy and its dysregulation during physiological and pathological aging, which is of particular interest for identifying pharmaceutical targets and developing potential therapies for neurodegenerative conditions.

## INTRODUCTION

The age-related alterations inevitably developing in the brain during aging pose a significant societal challenge, as they are frequently accompanied by the onset of cognitive impairment and underlie the pathogenesis of a number of neurodegenerative diseases [1, 2].

Mitochondria, organelles with a broad range of functions aimed at coordinating the intracellular homeostasis, play a particularly crucial role in maintaining adequate neuronal function upon the agerelated and pathological involution of the brain [3]. Mitochondrial dysfunction significantly increases the risk of one developing age-related neurodegenerative diseases because of the energy deficit that develops in nervous tissue, as well as the overproduction of reactive oxygen species, initiation of apoptosis and inflammatory responses, and the disruption to synaptic transmission [4].

The structural and functional characteristics of mitochondria are consistently under rapid transformation, their key stages being collectively known as “the mitochondrial dynamics.” Mitochondrial dynamics involve key processes such as biogenesis, fission, and fusion of these organelles, even as they also require an adequate system for their elimination known as mitophagy [5].

Mitophagy is a process that aims to dispose of damaged organelles and regulate the cellular content of mitochondria within the boundaries required for maintaining a metabolic balance [6]. It involves the swallowing of defective mitochondria by specialized vesicles, followed by their fusion with the lysosomes responsible for the degradation of defective organelles [7, 8, 9].

Mitophagy is critically important in maintaining a functional pool of neurons because of the unique structure and function of the nervous tissue, its voracious appetite for energy, and the need for a continuous renewal of the components of the cytoplasm.

Brain aging is accompanied by a decline in mitophagic activity, which aggravates mitochondrial dysfunction, and increases the risk of developing neurodegenerative diseases [10, 11]. According to current understanding, the accumulation of neurotoxic protein aggregates, which play a pivotal role in the pathogenesis of this pathology, is attributed to mutations in the genes coding for mitophagy-initiating proteins (PINK1, Parkin, and DJ-1) [12].

Despite the relevance and high societal significance of this issue, many aspects of brain aging remain insufficiently studied. Elucidating the role played by mitochondrial dysfunction and identifying the key markers of mitophagy in age-related involution are topical problems in modern gerontology and a muchneeded step in identifying novel pharmaceutical neurodegeneration targets.

## THE GENERAL DATA ON THE MECHANISMS OF MACROAUTOPHAGY. MITOPHAGY

Large intracellular substrates (aged and damaged organelles in particular) are removed through macroautophagy – the type of autophagy in which the identification and further degradation of defective structures take place within the autophagosome, which is formed via fusion of a lysosome and a phagophore, a double-membraned organelle. Autophagic processes within the cell are triggered by various factors such as the accumulation of pathological protein aggregates, exposure to hypoxia, nutrient deficiency, and oxidative stress. Numerous proteins encoded by autophagy-related genes (Atg) are involved in the perception of autophagy initiation signals and autophagosome formation [13]. The LC3 (ATG8) protein, which resides on the phagophore membrane and binds to a pre-ubiquitinated target via adaptor proteins, plays a special role in autophagosome degradation [14]. The best-studied autophagy adaptors include p62 (the key adaptor protein in nearly all mitophagy pathways), NBR1 (involved in peroxisome degradation), NDP52 (involved in ubiquitin-dependent mitophagy), as well as TAX1BP1 and optineurin (OPTN), which are required for ubiquitin-dependent mitophagy and the autophagy of protein aggregates [15].

The plasma membrane and cellular organelles (the Golgi complex, the endoplasmic reticulum, and mitochondria) are the potential sources of phagophore formation.

De novo assembly of the phagophore is initiated by two cytoplasmic protein complexes: PI3K (class III PI3K complex I) and the Atg1/ULK1 complex, which are comprised of catalytic and regulatory subunits [6, 16, 17]. Phosphorylation of the PI3K class III complex induces the local production of the membrane phospholipid PI3P (phosphatidylinositol 3-phosphate) in specialized endoplasmic reticulum subdomains known as omegasomes [18]. PI3P is needed in order to recruit the phospholipid molecules involved in phagophore growth via binding of the effector proteins WIPI and DFCP. These proteins mediate the interplay between PI3P and the two conjugation systems, LC3/ ATG7/ATG3 and ATG5/12/ATG16L1 [19]. At the next stage, autophagy-related (Atg) proteins are incorporated into the isolation membrane, resulting in phagophore formation [20, 21]. The conjugation systems are required not only for phagophore expansion, but also for the completion of autophagosome formation and cargo sequestration. Selective uptake of various targets is ensured by receptor proteins residing on the surface of an autophagy target through specialized autophagic adaptor proteins [22]. Despite their versatility, adaptors seem to utilize a common autophagy mechanism: recruitment of the ULK1/2 complex and binding to the FIP200 subunit (an adhesion protein) to initiate autophagosome formation [15, 23].

After substrate degradation in the autophagosome, macromolecules are released into the cytosol and they re-enter the metabolic processes in the cell [16, 24]. Autophagy is regulated by the two key signaling pathways:

(1) The PI3K/AKT/mTOR pathway inhibiting the autophagy and preventing autophagosome formation. The activity of mTORC1 (the mammalian target of rapamycin complex 1) is affected by the intracellular levels of amino acids, insulin, and growth factors. (2) The AMPK signaling pathway that responds to the ATP level and is activated under hypoxic conditions [16, 25].

The roles played by other signaling molecules such as sirtuins, TFEB (transcription factor EB), etc., in the autophagy mechanisms are less studied and require detailed investigation.

Phagophore assembly is also regulated by mitochondrial proteins. Thus, the well-known protein Beclin 1, a component of the pro-autophagic class III PI3K complex involved in phagophore assembly, initiates Beclin 1-dependent autophagy at the levels of both the endoplasmic reticulum and mitochondria [26, 27].

Another autophagy initiator, the protein endophilin B1, can be recruited to the outer mitochondrial membrane under stress conditions, where it activates the aforementioned class III PI3K initiation complex by binding to the adaptor protein Beclin 1 [26].

Mitophagy is the selective degradation of mitochondria via autophagosome processing. The mitophagy is preceded by changes in the mitochondrial morphology. Thus, mitochondrial fission mediated by the DRP1 and Fis1 proteins ensures the peripheral fragmentation of mitochondria, isolating the damaged segments of the organelle to be subsequently eliminated [28].

**Fig. 1:**
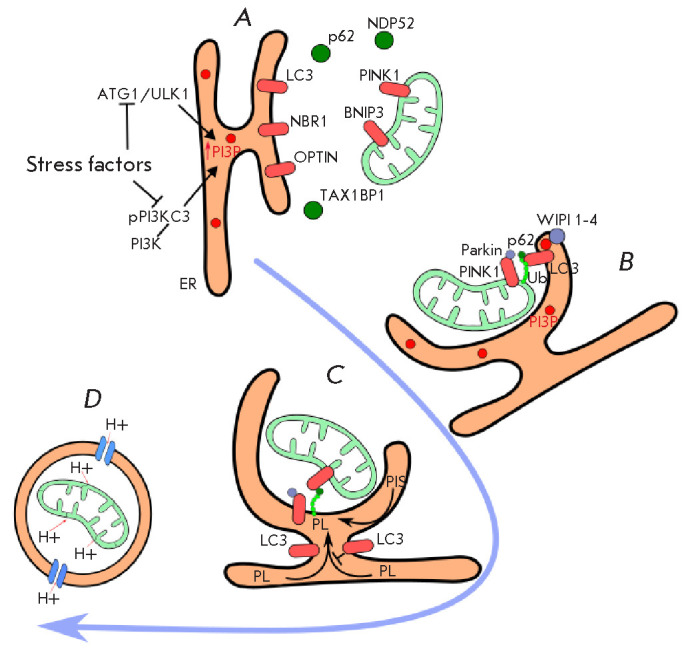
The mechanism of mitophagy. Stages: (A) mitophagy initiation; (B) receptor interactions; (C) phagophore growth; (D) vesicle–lysosome fusion. Mitophagy initiation occurs under the influence of stress factors and is accompanied by the activation of ATG1/ULK1 and phosphorylation of PI3K, which induces PI3P production in the ER. PI3P is required for the binding of the effector WIPI proteins that interact with the LC3 conjugation system. The selective mitochondrial uptake is implemented with the participation of specialized adaptor proteins (TAX1BP1, NBP52, p62, OPTIN, and NBR1) (A). Next, Parkin and ubiquitin-mediated LC3 binding to PINK1 on the mitochondrial membrane occurs. By joining to PI3P WIPI 1–4 ensures interplay with LC3 and proper functioning of the complex (B). Phagophore growth takes place through the transfer of PLs from the ER lumen, with the participation of PI3P. Simultaneously, PIS is activated in the phagophore walls, initiating de novo phospholipid synthesis (C). LC3 ensures vesicle cleavage from the ER. It merges with the lysosome, followed by the destruction of its contents (D). PI3K – phosphoinoside-3-kinase; PI3P – phosphatidylinositol-3-phosphate; TAX1BP1 – Tax1-binding protein 1; NBP52 – calcium-binding protein 2; OPTIN – optineurin; PINK1 – PTEN-induced kinase 1; BNIP3 – protein 3 interacting with protein BCL2; PL – phospholipids; PIS – phosphatidylinositol synthase; ER – endoplasmic reticulum

The mechanism of classical mitophagy is based on the induction of mitochondrial membrane PTENinduced putative kinase 1 (PINK1) and the Parkin protein (PARK2), a cytosolic E3 ubiquitin ligase. Hence, the PINK1 (PARK6) and PARK2 genes encoding the proteins associated with the familial Parkinson’s disease play a crucial role in mitochondrial quality control. In this case, the loss of the inner mitochondrial membrane potential accompanying damage to mitochondria is a signal for mitophagy activation. Known PINK1 substrates include ubiquitin and the ubiquitin-like domain of Parkin. Phosphorylation of these targets at a conserved serine residue (S65) induces Parkin activation, followed by absorbtion of damaged mitochondria and autophagosome formation [29] (Fig. 1).

Parkin translocation from the cytosol to the outer mitochondrial membrane is dependent on the PINK1 activity. In turn, Parkin catalyzes the ubiquitination and proteasomal degradation of various outer mitochondrial membrane proteins, including Drp1, Miro, and mitofusins 1 and 2 (MFN1/2). This mechanism blocks mitochondrial fusion, making it possible to isolate damaged organelles and initiate autophagy via a system of adaptor proteins.

Under hypoxic conditions and exposure to various toxic agents, mitophagy can proceed via a PINK1– Parkin-independent pathway through the following mitochondrial membrane receptors containing LIR motifs:

• The proteins AMBRA1, BNIP3, FUNDC1, and NIX on the outer mitochondrial membrane;

• Cardiolipin and PHB2 on the inner mitochondrial membrane.

Ubiquitination of these receptors is a signal for the cargo receptors p62/SQSTM1, NDP52, optineurin, etc., which bind to ubiquitin and the autophagosomal membrane protein LC3B, thereby mediating the mitophagy [30].

## MITOPHAGY DURING PHYSIOLOGICAL AGING

As confirmed by electron microscopy studies, mitochondrial disorganization that progresses with aging is accompanied by mitochondrial dysfunction [31].

Research into the mitochondrial ultrastructure during physiological aging revealed a reduction in the length and surface area of mitochondria, along with changes in cristae and membranes. These morphological changes were shown to correlate with an upregulated expression of phosphorylated Drp1, a marker of mitochondrial fission, as well as reduced levels of the mitochondrial fusion protein Mfn2 and the autophagy marker LC3B. The increased fragmentation of mitochondria observed during aging alters their function, including a reduction in ATP/ADP transport due to reduced levels of the VDAC1 protein (involved in the regulation of mitochondrial membrane permeability), as well as a greater severity of oxidative damage. Defective mitochondria are characterized by rupture of the outer membrane and release of apoptogenic factors into the cytoplasm, followed by cell death. The aforementioned age-related morphofunctional modifications of organelles reduce neuronal density and exacerbate neurodegeneration [3].

Numerous studies prove that autophagy intensity progressively decreases during age-related involution and in age-related diseases [32, 33, 34, 35, 36, 37].

The use of the mt-Keima probe (a monomeric acid-stable fluorescent protein with an affinity to the mitochondrial matrix) for quantifying mitophagy in a transgenic mouse line revealed age-related reduction of the mitophagy levels in neurons in the hippocampal dentate gyrus [33]. Overexpression of the key markers of PINK1–Parkin-dependent mitophagy in aging models was found to be accompanied by longer lifespans in the model organisms (Drosophila melanogaster and Caenorhabditis elegans) [34]. An elevated Parkin level, both in the brain tissue and cerebral vessels, was revealed in a group of old mice aged 24 months in [35, 36]. Upregulated Parkin expression was found to reduce the number of point mutations in mitochondrial DNA that cause mitochondrial dysfunction in [37].

It has been demonstrated using cellular models that neuroapoptosis decreases in the absence of PINK1, confirming the role played by this protein in neuronal survival during aging [38].

**Fig. 2:**
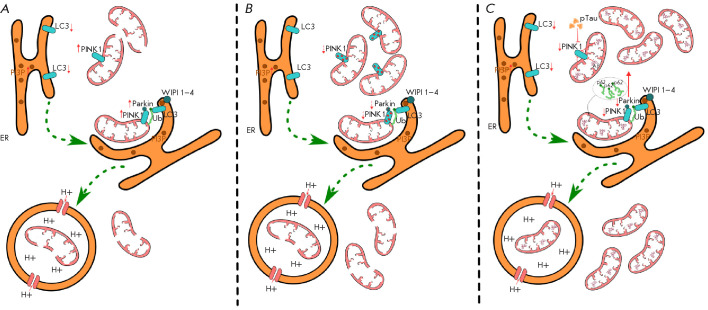
Changes in the mitophagy process at the initiation stage and receptor interactions upon physiological aging and neurodegenerative diseases. (A) Aging. Characterized by the accumulation of defective mitochondria and mitophagy dysfunction. Progressive mitochondrial disorganization is accompanied by a compensatory increase in the PINK1 and Parkin levels. Reduced LC3 expression disrupts the interaction between phagophore receptors and mitochondria, leading to the inhibition of mitophagy. (B) Parkinson’s disease. Accompanied by decreased utilization of mitochondria. In genetic forms of PD, mutations are detected in the genes encoding the PINK1 and Parkin synthesis, leading to inactivation of the respective proteins. (C) Alzheimer’s disease. Characterized by a significant increase in the pool of defective mitochondria and reduced intensity of mitophagy. The accumulation of abnormal protein aggregates contributes to mitochondrial damage, reduces the PINK1 and Parkin levels, and increases the LC3 and p62 levels. PI3P – phosphatidylinositol-3-phosphate; p62 – ubiquitin-binding protein p62. ┴ – mediated effect

Memory loss during aging has been found to correlate with the downregulated expression of the Mfn1, Mfn2, Opa1, LAMP2, and LC3 genes, while PINK1 and Parkin expression is upregulated, which affects the mitochondrial membrane potential. These changes in the dynamics of LAMP2, LC3, PINK1, and Parkin expressions are indicative of mitophagy dysfunction [3] (Fig. 2).

There is a growing body of evidence showing that physical activity effectively induces autophagy, alters mitochondrial dynamics to keep them functioning, and has a neuroprotective effect. Different types of physical exercises can induce autophagy in the cerebral cortex of young and adult animals and mitigate autophagic dysfunction in the aged brain. Recent studies have revealed that physical training elevates the levels of the autophagy-related proteins LC3-II/LC3-I, LC3-II, p62, Atg7, Bnip3L, and Parkin, as well as the Mfn2 and Drp1 levels [39]. Furthermore, Liu et al. in [33] demonstrated how strenuous physical exercise induced PINK1-dependent mitophagy in mt-Keima mice.

Hence, the balance between the mitochondrial dynamics and mitophagy is a specific compensatory mechanism playing a pivotal role in maintaining the stability of the functioning of these organelles in the aging brain.

## MITOPHAGY IN NEURODEGENERATIVE DISEASES

Mitophagy plays a critical role in the pathogenesis of neurodegenerative diseases such as Alzheimer’s disease (AD) and Parkinson’s disease (PD), whose risk increase significantly with age [2].

Mitochondrial dysfunction and oxidative stress were found to be central pathogenic factors in genetically determined forms of Parkinson’s disease [40]. The early-onset forms of PD can be caused by mutations in the PARK2 (Parkin), PINK1, and DJ-1 genes, which code for proteins residing in mitochondria (Fig. 2). The loss of these proteins increases the susceptibility to oxidative stress and disrupts the energy metabolism [41]. INK1 overexpression was shown to inhibit the translation of DRP1 mRNA and reduce its translocation from the cytosol to the mitochondrial surface, thus causing the formation of elongated chain-linked mitochondria and impeding the elimination of damaged organelles. PINK1-mediated ubiquitination of DRP1 results in its proteasomal degradation followed by inactivation, thus also reducing the intensity of mitochondrial fission [42]. Meanwhile, PINK1 knockdown increases mitochondrial fragmentation [43].

Since PINK1 is the only known kinase that catalyzes ubiquitin phosphorylation, the detection of ubiquitin phosphorylated at S65 can be used to assess PINK1 activity and is viewed as a biomarker of mitochondrial stress and autophagy [44]. Mitochondrial damage leads to PINK1 accumulation because of its impaired degradation by the PARL (presenilin-associated rhomboid-like protein) protease residing on the mitochondrial inner membrane [45]. Unlike for idiopathic Parkinson’s disease, Lewy bodies sometimes are not detected in substantia nigra neurons in postmortem specimens collected from patients carrying PINK1 or Parkin mutations [46]. This is presumably caused by the involvement of PINK1 and Parkin in the long-term survival of dopaminergic neurons, and disruption of this process results in their rapid death, without the accumulation of pathological proteins, which is supported by PINK1 knockdown experiments.

Research has demonstrated that the PINK1– Parkin-independent pathway involving cardiolipin also has defects [47]. Neurons carrying the SNCA mutation typical of Parkinson’s disease are characterized by a more intense cardiolipin translocation to the outer mitochondrial membrane. In turn, this phospholipid, capable of refolding α-synuclein fibrils, enhances mitophagic flux by interacting with LC3 on mitochondria, thus leading to mitochondrial dysfunction, which is further complicated by defects in mitophagy. At early stages of PD, synaptic mitochondria lose their cardiolipin cluster, thus reducing the intensity of mitophagy [47, 48].

A number of studies have demonstrated that mitochondrial deubiquitinase (USP30) can be a promising target for maintaining mitophagy in patients with Parkinson’s disease. Reduction of the USP30 levels in various models of this disease has been shown to optimize mitochondrial function [5, 49, 50].

The mitochondrial dynamics and mitophagy are also impaired during the development of Alzheimer’s disease (Fig. 2). This is evidenced by alterations in the expression of the ATG5, Beclin1, LC3A, LC3B, PINK1, TERT, BCL2, and BNIP3L genes detected in a mouse model of AD [51].

A 30–50% reduction in the basal mitophagy level was observed in the hippocampus of AD patients, accompanied by the accumulation of damaged mitochondria characterized by reduced size, disorganized cristae, and decreased ATP production [52]. Elevated PINK1 levels were detected in the hippocampus of patients with early-stage AD, while Parkin levels were increased at its late stages, which is indicative of impaired mitophagy because of defective initiation of the PINK1/Parkin-dependent pathway [45]. Impaired recruitment of activated LC3 to phagophore membranes, dysfunction of the AMPK signaling cascade, and disrupted fusion of mitophagosomes with lysosomes have also been observed [52].

An increased p62 level, an elevated LC3II/LC3I ratio, and a reduced PINK1 level were observed in mitochondrial fractions isolated from the brains of patients with late-stage AD, which is also indicative of mitophagy failure [53]. The accumulation of pathological protein aggregates in Alzheimer’s disease significantly affects the mitochondrial dynamics and mitophagy. Thus, intraventricular administration of β-amyloid in rats reduced the PINK1, Parkin, and BCL-1 levels, while increasing the hippocampal level of p62 in [54]. The accumulation of total and phosphorylated tau protein is accompanied by an increase in the mitochondrial membrane potential, preventing PINK1 stabilization on the outer mitochondrial membrane and impeding Parkin recruitment. The reduced PINK1 content on the outer mitochondrial membrane suppresses the activation of Parkin and E3 ubiquitin ligase, thus disrupting the further stages of autophagy and mitophagy [55]. Parkin overexpression restores mitophagy and the mitochondrial membrane potential [56]. Alterations in the mitochondrial dynamics accompanying the development of AD involve enhanced organelle fission. The accumulation of toxic tau protein and β-amyloid increases DRP1 phosphorylation and promotes its translocation into mitochondria [57]. Mitochondrial hyperfragmentation ultimately triggers cell death and neurodegeneration.

Sukhorukov et al. [11] suggested that alterations in ATP and NAD^+^ homeostasis can be among the reasons behind impaired mitophagy in AD, which was supported by the fact that a reduced intracellular NAD^+^ level initiates the aggregation of misfolded proteins, promoting defective autophagy, followed by neuronal death.

The activity of two neuroprotective genes, Sirtuin1 (SIRT1) and Sirtuin3 (SIRT3), which encode the synthesis of eponymous proteins, is also reduced in AD. Sirtuin-1 functions to induce autophagy/mitophagy via deacetylating the ATG5, ATG7, and ATG8/LC3 proteins. Moreover, sirtuin-1 stabilizes PINK1 and increases the levels of LC3 and Nix/BNIP3, which are involved in mitophagy [58]. In turn, sirtuin-3 activates the FOXO3 gene regulating apoptosis and autophagy [59].

The altered dynamics of lysosomal activity, which is typical of the pathogenesis of AD, stems from a deficiency of the lysosomes in brain tissue. In turn, this disrupts the clearance of autophagic aggregates and is also believed to cause defective mitophagy. Thus, in the hereditary form of AD, mutations in the PSEN1 gene encoding presenilin 1 cause hyper-alkalinization of the lysosomal environment, pathological reduction in lysosomal hydrolase activities, and a rise in p62 levels [56].

Many diseases, including neurodegenerative disorders, are characterized by excessive accumulation of advanced glycation end-products which induce oxidative stress and inflammation by generating reactive oxygen species. In turn, reactive oxygen species are considered a primary factor in the triggering of stress-induced mitophagy. Upregulated expression of the receptor for advanced glycation end-products was detected in post-mortem brain specimens from AD patients [60, 61].

Hence, although the involvement of the PINK1– Parkin-dependent pathway in mitophagy mechanisms and its role in the pathogenesis of neurodegenerative diseases have been studied relatively well, a number of questions remain open. Much focus has recently been directed at investigating alternative mitophagy pathways such as the degradation of mitochondrial components via mitochondrial-derived vesicles containing oxidized proteins, lipids, mutant mitochondrial DNA, and reactive oxygen species [43]. A link between mitochondrial-derived vesicles, mitophagy defects, and autoimmune responses that cause neuronal death in Parkinson’s disease has recently been discovered [62].

## CONCLUSIONS

Mitophagy plays a pivotal role in maintaining physiological homeostasis, the aging mechanisms, and the pathogenesis of neurodegenerative disorders. Various molecules that modulate mitophagic activity in nervous tissues are currently under study as potential candidates for developing therapeutics against neurodegenerative diseases. Meanwhile, given the diversity of the regulatory pathways of mitophagy, there is no question that this list of candidates will expand due to the multiple factors that are indicative of the state of mitophagy in specific types of nervous tissue cells in response to various stressors.

Overall, regardless of the existing interest in the role of mitophagy in age-related involution and the pathogenesis of age-related diseases, the mechanisms through which it affects the organismal aging remain insufficiently studied. The range of questions that need to be resolved includes the involvement of various regulatory signaling molecules in coordination with inter-organelle interactions, the specific features of the mitochondrial dynamics preceding the mitophagy, and the mechanisms of autophagosome degradation under mitochondrial stress. Particular focus should be placed on the mechanisms of initiation (activation) of both classical and receptor-mediated autophagy.

Hence, further research into the interplay between potential key markers of mitophagy and their relative contribution to neurodegeneration is of extreme importance for identifying novel promising pharmaceutical targets.

## Abbreviations

AD: Alzheimer’s disease
PD: Parkinson’s disease
